# Sepsis Induces Specific Changes in Histone Modification Patterns in Human Monocytes

**DOI:** 10.1371/journal.pone.0121748

**Published:** 2015-03-20

**Authors:** Sebastian Weiterer, Florian Uhle, Christoph Lichtenstern, Benedikt H. Siegler, Sabin Bhuju, Michael Jarek, Marek Bartkuhn, Markus A. Weigand

**Affiliations:** 1 Department of Anaesthesiology, Heidelberg University Hospital, Heidelberg, Germany; 2 German Centre for Infection Research (DZIF), partner site Giessen-Marburg-Langen, Giessen, Germany; 3 Genome Analytics, Helmholtz Centre for Infection Research, Braunschweig, Germany; 4 Institute for Genetics, Justus-Liebig-University, Giessen, Germany; National Institutes of Health, UNITED STATES

## Abstract

**Background:**

Sepsis is a global burden and the primary cause of death in intensive care units worldwide. The pathophysiological changes induced by the host’s systemic inflammatory response to infection are not yet fully understood. During sepsis, the immune system is confronted with a variety of factors, which are integrated within the individual cells and result in changes of their basal state of responsiveness. Epigenetic mechanisms like histone modifications are known to participate in the control of immune reactions, but so far the situation during sepsis is unknown.

**Methods and Findings:**

In a pilot approach, we performed combined chromatin immunoprecipitation followed by high-throughput sequencing to assess the genome-wide distribution of the chromatin modifications histone 3 lysine 4 and 27 trimethylation and lysine 9 acetylation in monocytes isolated from healthy donors (n = 4) and patients with sepsis (n = 2). Despite different underlying causes for sepsis, a comparison over promoter regions shows a high correlation between the patients for all chromatin marks. These findings hold true also when comparing patients to healthy controls. Despite the global similarity, differential analysis reveals a set of distinct promoters with significant enrichment or depletion of histone marks. Further analysis of overrepresented GO terms show an enrichment of genes involved in immune function. To the most prominent ones belong different members of the HLA family located within the MHC cluster together with the gene coding for the major regulator of this locus—CIITA.

**Conclusions:**

We are able to show for the first time that sepsis in humans induces selective and precise changes of chromatin modifications in distinct promoter regions of immunologically relevant genes, shedding light on basal regulatory mechanisms that might be contributing to the functional changes occurring in monocytes.

## Introduction

Sepsis is a global burden and the primary cause of death on ICUs all over the world [1–3]. During sepsis, the immune system is confronted with a variety of factors, which are integrated within the individual cells and result in changes of their basal state of responsiveness. Exuberant activation of immune cells is combined with a release of proinflammatory cytokines and simultaneously compensatory mechanisms to counterbalance the generalized inflammatory reaction, involving high levels of antiinflammatory mediators [4]. The compensatory reaction of the immune system seems to often dominate the response, resulting in a prolonged state of sepsis-induced immunosuppression [5]. Despite the knowledge that epigenetic mechanisms like e.g. histone modifications participate in the control of the immune system [6], the pathophysiological changes induced by the host’s systemic inflammatory response to an infection are yet not fully understood.

Histones can be posttranslationally modified by the enzyme-catalyzed addition of chemical groups to their N-terminal tails, e.g. acetylation, phosphorylation or methylation. The specific presence or absence of these histone modifications in promoter regions is functionally correlated with the expression of the associated genes in defined genomic regions [7]. Trimethylation (me3) of lysine (K) 27 histone (H) 3 (H3K27me3) has been detected to be enriched at promoters of genes with repressed transcriptional activity, while trimethylation of K4 and acetylation (ac) of K9 of H3 are known as markers of active or poised promoter regions.

By influencing gene expression, histone modifications seem to be indirectly associated with the regulation of different kinds of cell functions. Therefore, also the regulation of histone modifying enzymes like histone deacetylases (HDACs) seems to play a key role in inflammatory gene expression. It has been demonstrated that HDAC3-deficient murine macrophages lack the ability to express inflammatory genes after LPS (lipopolysaccharide) stimulation, which is attributable to a secondary effect by the loss of LPS-induced IFN-β (interferone-β) expression [8]. Moreover, LPS stimulation induces gene expression changes in murine bone marrow-derived macrophages by regulating several members of the histone deactylase family [9]. It affects proinflammatory gene expression by induction of histone deacetylases HDACs -4, -5 and -7 after transient repression and has a rapidly inducing effect on HDAC-1 mRNA.

Besides manipulation of acetylation through histone deacetylases, also changes in methylation have been found to be important during immune reactions. The histone demethylase Jmjd3 (jumonji domain containing 3) has been identified to regulate immune response in murine macrophages after induction by the transcription factor NF-kB [10]. It removes H3K27me3, a histone modification which is highly associated with repressed promoter regions. Jmjd3 also interacts with H3K4me3 associated active promoter regions as well as RNA polymerase II. Overall Jmjd3 contributes largely to the transcriptional output of LPS-activated macrophages.

This is in line with the finding, that continuous interleukin-4 stimulation induces an increase in Jmjd3 expression and decreases H3K27 methylation at the promoter regions of alternative macrophage marker genes followed by an increased transcriptional activation of these genes [11].

Animal experiments using the cecal ligation and puncture (CLP) model, mimicking the clinical condition of polymicrobial peritonitis, showed both a decreased H4ac and H3K4me3 with reduced recruitment of RNA polymerase II and associated with decreased cytokine gene expression in lung macrophages. These findings correlated with the induction of Interleukin-1 receptor-associated kinase-M, a negatively regulating protein of the TLR signaling pathways. Interestingly, only few genes showed a transiently reduced expression in response to LPS during sepsis [12].

Despite all evidence from animal experiments, the global pathophysiological mechanisms are still unknown and the mounting evidence that genomic responses in mouse models only poorly mimic the situation during human inflammatory diseases raises the ultimate need for primary cell analysis from patients suffering from sepsis [13].

Monocytes are key players in the innate immune system and their cellular functions are of high importance during early immune reactions. During sepsis, the classical monocyte subpopulation (CD14^++^ CD16^−^) seems to play an important part in the initial pro-inflammatory defence mechanism [14]. In fact, functional monocyte regeneration seems to shorten time of mechanical ventilation as well as intrahospital and intensive care unit stay [15]. Studies including adult and pediatric patients with sepsis provide evidence, that substitution with GM-CSF, a cytokine involved in myeloid hematopoiesis and the modulation of cell responsiveness, might lead to a beneficial reduction of nosocomial infection [16–17].

Despite all efforts in immune and sepsis research the exact mechanisms leading to the pathological monocyte dysfunction are still unknown. An analysis of epigenetic modifications of human immune cells may help to understand the genomic alterations occurring and therefore unravel novel pathophysiological mechanisms in patients suffering from sepsis.

In order to set the stage for epigenomic studies in the future, we conducted genome-wide histone modification analysis of human CD14++ CD16- monocytes from two patients suffering from sepsis. Our study demonstrates for the first time that sepsis in humans does not result in an uncontrolled remodeling of the chromatin landscape, but in selective and precise changes of chromatin modifications in distinct promoter regions of immunologically relevant genes.

## Results and Discussion

To assess the genome-wide distribution of the chromatin modifications H3K4me3, H3K27me3 and H3K9ac in CD14++ CD16- monocytes from patients suffering from sepsis (for characterictics see S1 Table), we isolated blood monocytes and subjected them to chromatin immunoprecipitation and high-throughput sequencing in a pilot approach. To detect differential changes, the patient datasets were compared to previously generated control datasets of healty volunteers acquired with the same workflow as described earlier [18].

**Fig 1:**
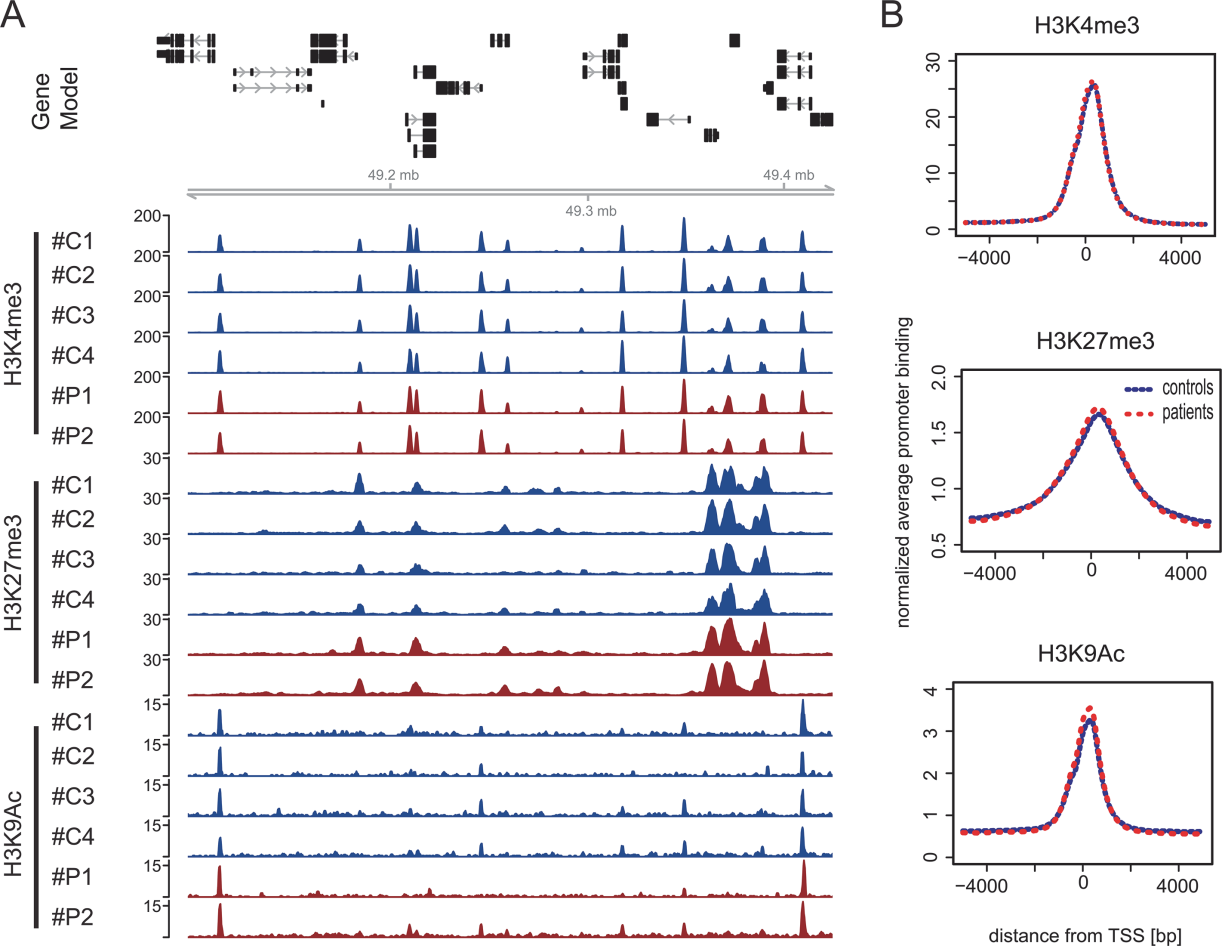
ChIP-seq profiles for three histone modifcations in CD14++ CD16- monocytes from healthy control probands and patients suffering from sepsis. A) Genome browser snapshot of a region from chromosome 12 (chr12:102,282,711–102,874,166): Top panel shows the gene models for a randomly chosen genomic location, indicating that signals observed for individual histone modifications in most cases localize to the promoter regions of genes and are indifferent from each other in independent samples. For two patients (red; #P1 and #P2) and four controls (blue; #C1-#C4) we show normalized ChIP-seq coverage vectors for 3 modifications (H3K4me3, H3K27me3 and H3K9ac). B) Normalized average histone modification profiles across all annotated RefSeq transcription start sites indicate promoter specific association of histone marks. After appropriate normalization, ChIP-seq binding profiles for H3K4me3, H3K27me3 and H3K9ac show on genome-wide level a very similar distribution pattern when comparing patient (red) and control (blue) chromatin.

Randomly chosen genomic locations show an indifferent distribution of the histone modifications H3K4me3, H3K27me3 and H3K9ac between healthy donors and patients with sepsis with a clear association to the promoter regions of many genes. For visualization of this finding, a snapshot of an example region on chromosome 12 is depicted (Fig. 1A). Morever, an analysis of average histone modification profiles across all annotated RefSeq transcription start sites for H3K4me3, H3K27me3 and H3K9ac is able to confirm the highly similar distributions of patients and healthy volunteers (Fig. 1B). Overall, normalized global ChIP-seq binding profiles of chromatin prepared from CD14++ CD16- monocytes from septic patients indicate a similar distribution as seen with binding profiles of monocytes originating from healthy subjects.

**Fig 2:**
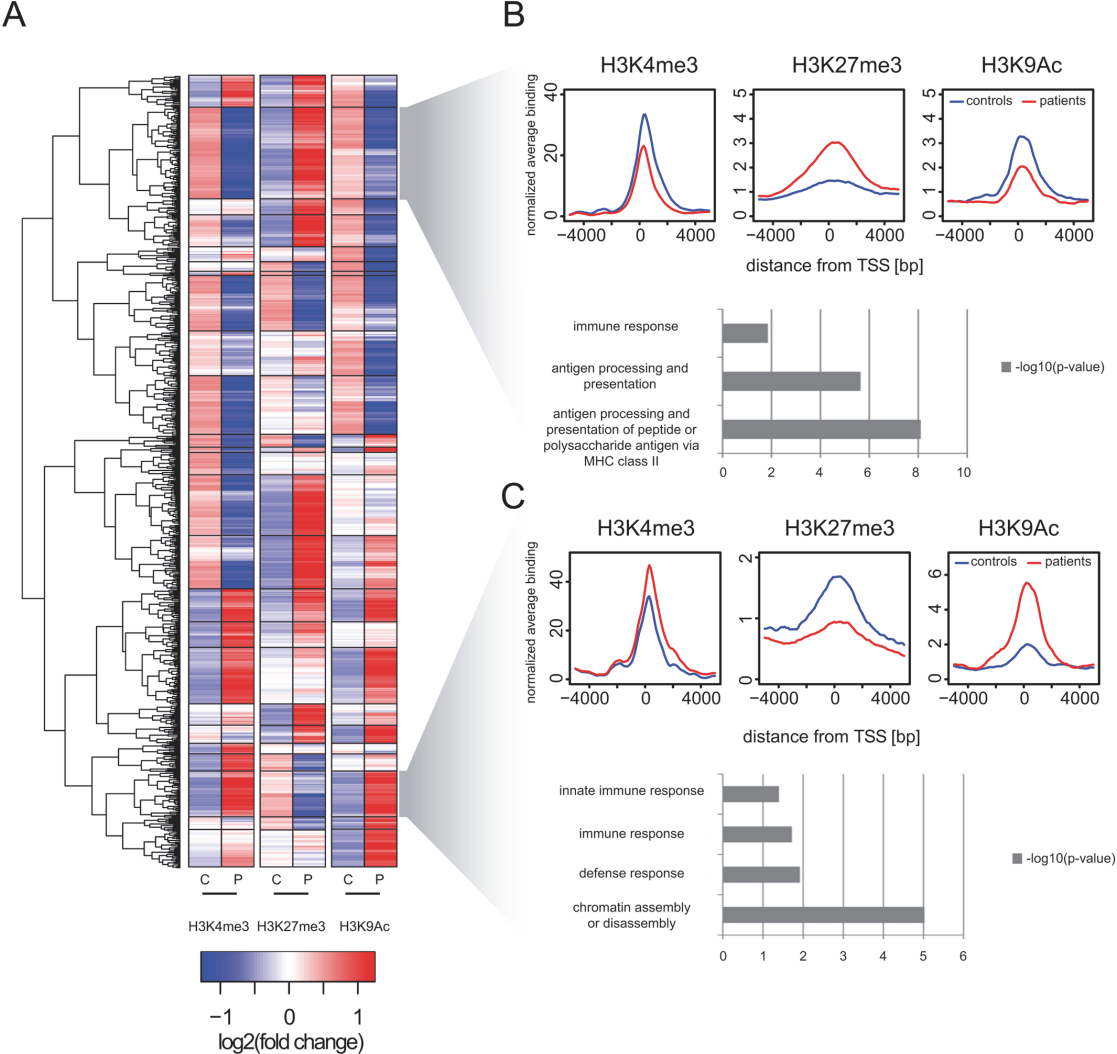
Cluster analysis of differential histone modification levels at transcriptional start sites reveals groups of co-regulated genes associated with immune-relevant functions. A) Heat map indicates result of un-supervised hierarchical clustering for 743 gene promoters with significant changes of at least one histone modification (absolute log2 (fold change) > = 1 and p<0.01). Depicted are the average z-scaled read counts (P: two patients; C: four controls) from a 2000 bp interval around the transcriptional start sites of RefSeq annotated transcripts. Rows indicate the data for the 743 genes. Dendrogram shows the relationship between individual genes. In total we identified 25 different clusters (separated by black boxes), exemplarily two clusters are indicated (grey shaded trapezoid). B) When comparing patients and controls the upper cluster (consisting of 86 genes) is characterized by decreased H3K4me3 and H3K9Ac binding as increased H3K27me3 binding (“silenced cluster”). Promoter specific differences are indicated by average histone modification profiles across all genes constituting the cluster (average binding shown; red: two patients; blue: four controls). The cluster is enriched for genes of the MHC complex and accordingly we identify related GO-terms to be significantly enriched for the genes of this cluster (bar plot shows -log10-transformed p-values derived from hypergeometric testing). C) When comparing patients and healthy controls the lower cluster (consisting of 43 genes) is characterized by increased H3K4me3 and H3K9ac binding and decreased H3K27me3 binding (“active cluster”). Promoter specific differences are indicated by average histone modification profiles across all genes constituting the cluster (average binding shown; red: two patients; blue: four controls). The cluster is enriched for genes related to immune response (bar plot shows -log10-transformed p-values derived from hypergeometric testing). In addition the cluster is enriched for genes related to the GO-term "chromatin assembly/ disassembly".

Despite the overall similarity in the chromatin phenotype of the promotor regions, we identified a group of 743 genes which exhibited a significantly changed coverage (fold change > = 2 with p< = 0.05) of their promoter regions for at least one of the examined posttranslational modifications (Fig. 2). Employing unsupervised hierarchichal clustering based on log2 transformed changes of respective modications in promoter regions, 743 genes were found to group into 25 clusters (Fig. 2A, for raw data see S2 Table). Interestingly, nearly all combinations of coverage changes of the modifications could be observed—ranging from clusters with a “poised” phenotype of enriched active as well as inactive modifications, to clusters lacking certain modifications towards clusters showing an opposed pattern of active and inactive marks. Two larger clusters of the latter type were subjected to further analysis (Fig. 2B+C). Compared to healthy controls, the 86 genes grouped into the upper cluster were found to exhibit a decreased presence of the active marks H3K4me3 and H3K9Ac in their promoter regions, while the inactive mark H3K27me3 was strongly enriched („silenced cluster“, Fig. 2B, for gene list see S3 Table). Overall, the resulting modification pattern hints towards a silencing of these genes in septic monocytes. To shed light on potential functional consequences of these alterations, GO term analysis was conducted and brought up a significant overrepresentation for genes involved in “immune response” and “antigen processing and presentation”. In contrast, the 43 genes in the “active cluster” revealed an inverted chromatin modification pattern with induced appearance of active chromatin marks and a decrease of H3K27me3 binding, suggesting a switch to an active promoter conformation and potentially induced gene expression (Fig. 2C, for gene list see S3 Table). Surprisingly and similar to the silenced cluster, GO term analysis found again an overrepresentation of genes involved in e.g. “immune response”. In addition, genes involved in “chromatin assembly and disassembly” were also present, although we currently have no understanding about how these genes may be involved in the process of septic disease. Taken together, the results are symbolic for the complex pathophysiology of sepsis which makes it difficult to define e.g. inflammatory genes *per se* as “bad” or “good”, as their functions in the systemic context might be interwoven with several other effector molecules and mediators. Nevertheless, our data indicate that the observed regulatory epigenetic mechanisms may happen in a precise and specialized manner and might not be a result of a global rearrangement of the chromatin landscape in monocytes. Increasing the knowledge about the target genes affected during sepsis might set the stage for the identification of upstream factors involved in mediating the observed changes, yielding eventually important therapeutic targets for the future.

**Fig 3:**
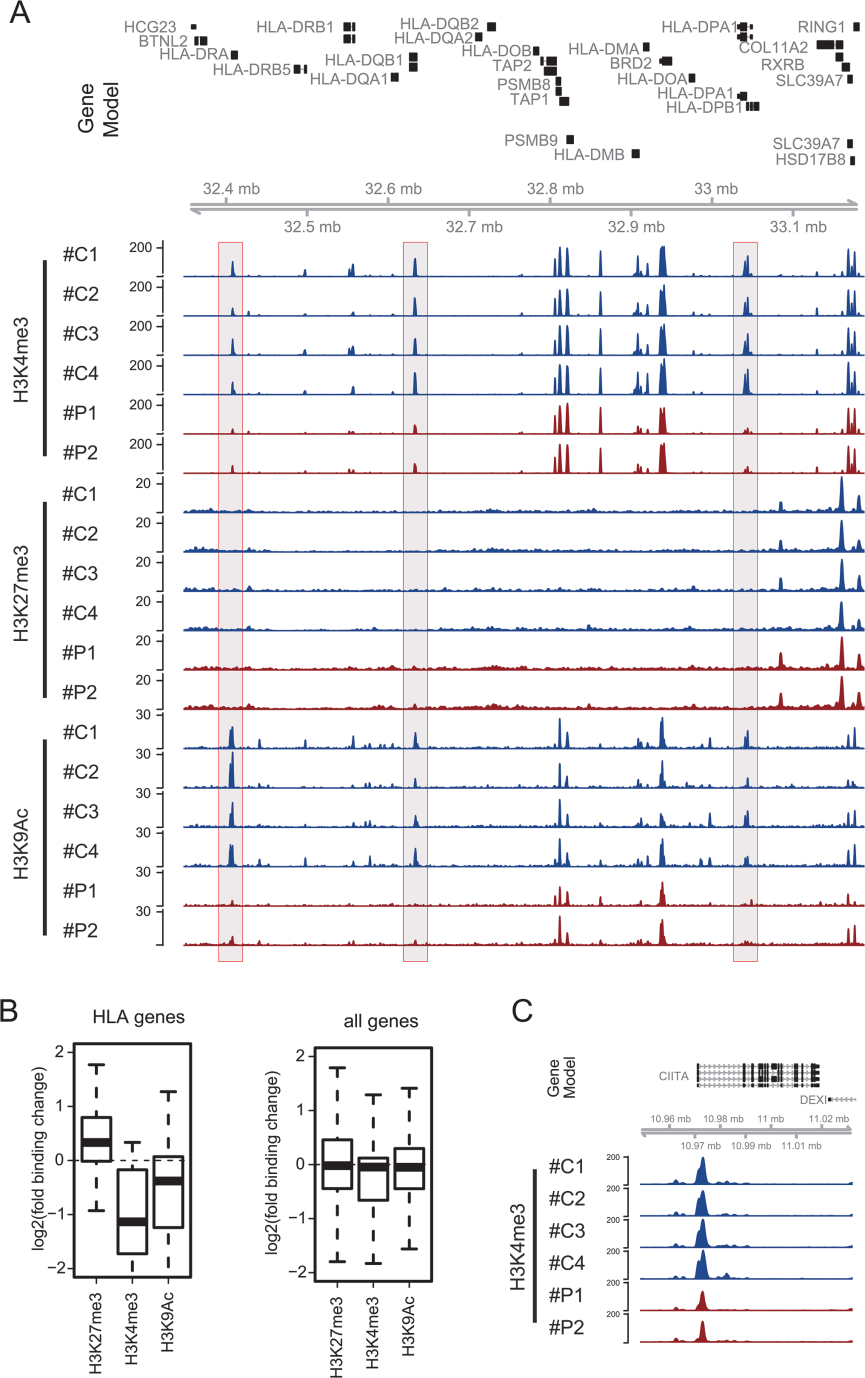
Systematic epigenetic regulation of MHC class II genes. A) Genome browser snapshot of a 841,000 bp region from the MHC class II locus located on chromosome 6 (6:32,347,232–33,188,204): Top panel shows the gene models. For two patients (red; #P1 and #P2) and four controls (blue; #C1-#C4) we show normalized ChIP-seq coverage vectors for 3 modifications (H3K4me3, H3K27me3 and H3K9ac). We indicate three regions (grey filled boxes) showing a clear reduction of active marks (H3K4me3 and H3K9Ac). B) Boxplots show log2-transformed average binding changes for three modifications for all MHC genes (left) in comparison to all genes as control (right). C) Genome browser snapshot of an 80,000 bp region from the CIITA gene locus: Top panel shows the gene models. In comparison between patients and controls we identified reduced H3K4me3 binding across the promoter region of the CIITA gene.

Monocytes belong to the group of antigen-presenting cells. Antigen-presentation is a crucial process during early immune responses required for full activation of the host response. Upon uptake, microorganisms are digested within the phagolysosomes and peptides are embedded into MHC class II complexes and presented on the surface of the cell for recognition by CD4+ T helper cells. During late sepsis a diminished expression of monocytic HLA-DR, which is a member of the MHC class II molecules, is a well known characteristic and closely correlates to the global loss of monocyte function, ultimately leading into an immuneparalytic state of the host [19]. The genes coding for the structural components necessary for the antigen presentation are all located within the Major Histocompatibility Complex (MHC) locus on chromosome 6p21.3 [20]. As we found an association of an inactive promoter phenotype with genes functionally annotated for antigen processing and presentation (Fig. 2A), we questioned which chromatin alterations might occur within this locus. In fact, our two patients with sepsis consistently exhibited a loss of active chromatin marks in several distinct promoter regions throughout the class II region of the locus, while changes in H3K27me3 occurred only subtle (Fig. 3A). The combined analysis of all HLA genes confirmed the visual impression indicating a systematic loss of H3K4me3 and H3K9ac and a slight gain of H3K27me3 binding across all HLA gene promoters (Fig. 3B). The same trend can be appreciated when analyzing the average HLA promoter read counts across the individual control and patient samples (S1 Fig.). Regulation of expression of the MHC class II genes depends on a “master regulator”, which has been identified as the transcriptional co-activator called CIITA [21]. It has been shown to associate with DNA-binding factors like e.g. cAMP response element binding protein (CREB) binding to the promoter regions of MHC class II genes, but which is not capable to induce gene expression by itself [22]. By recruiting the common gene expression machinery involving the polymerase II and the according co-factors, CIITA is able to induce expression. Moreover, CIITA has been shown to interact with CTCF, a factor involved in the global organization of chromatin and also to recruit chromatin remodeling factors [23]. Interestingly we found that the CIITA promoter displays a marked reduction of H3K4me3 when comparing sepsis patients versus controls (Fig. 3C). CIITA is thought to play a major regulatoy role in the regulation HLA genes in the MHC cluster. Therefore we speculate that down-regulation of HLA-DR as displayed by reduced H3K4me3 and H3K9ac levels may be functionally dependent on reduced activity of the CIITA gene promoter. Considering these earlier findings in combination with our own results, we hypothesize that the observed changes in the chromatin landscape of the locus might be the consequence of a lack of CIITA.

Overall, our results indicate changes in chromatin modifications, which seem to appear in a very specialized manner. As an example of high interest in sepsis, we found alterations in the MHC class II locus and its corresponding master regulator CIITA. In line with results of earlier studies in monocytes from septic patients, which show a decreased expression of MHC II genes as well as the gene encoding CIITA [24], our findings shed for the first time light on the basal regulatory mechanisms and point out the importance of the chromatin as regulatory player.

**Fig 4:**
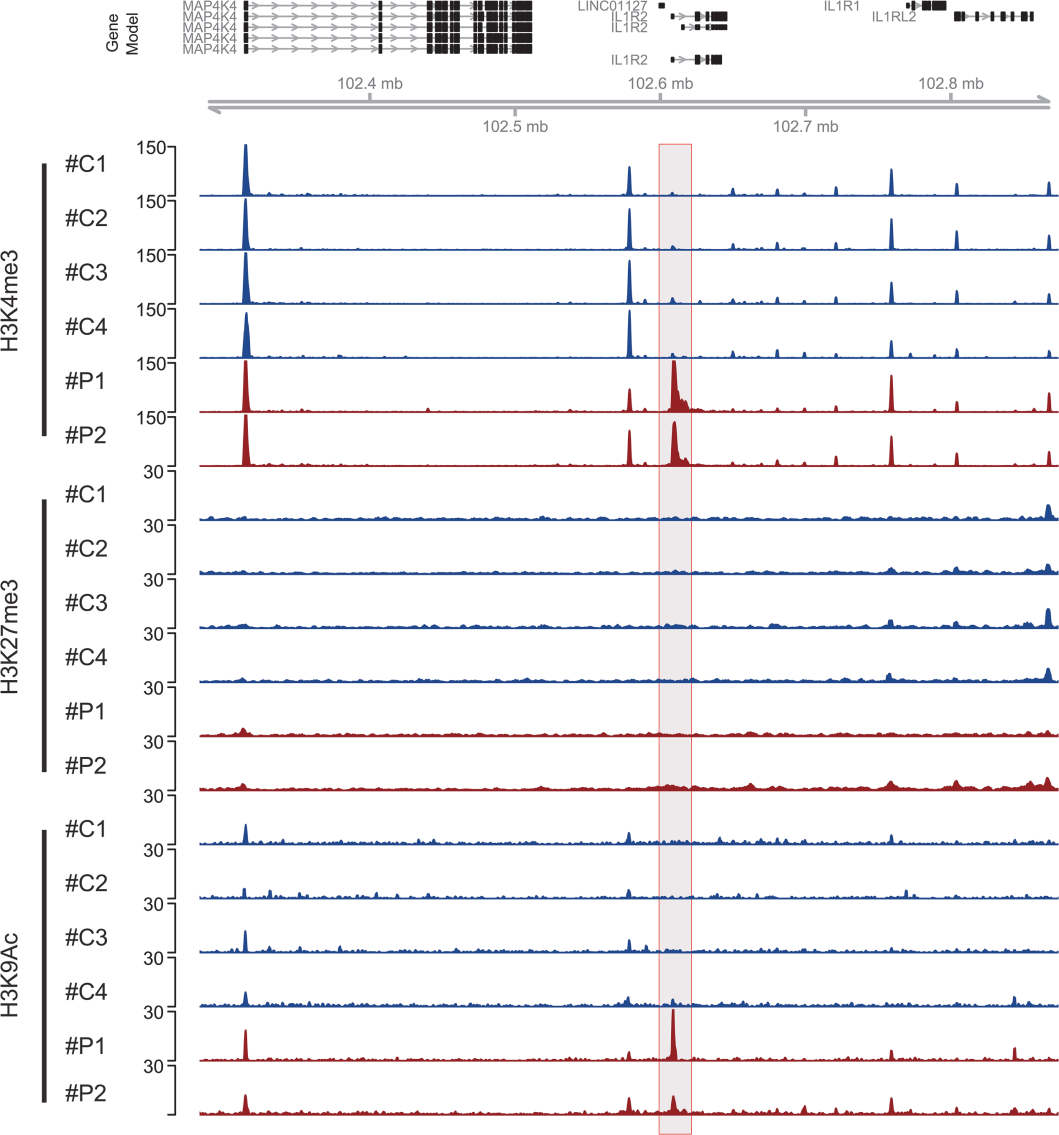
Chromatin changes in the promoter region of the IL1R2 gene. Genome browser snapshot of a 591,455 bp region of chromosome 2 (102,282,711–102,874,166): Top panel shows the gene models. For two patients (red; #P1 and #P2) and four controls (blue; #C1-#C4) we show normalized ChIP-seq coverage vectors for 3 modifications (H3K4me3, H3K27me3 and H3K9ac). We indicate three regions (grey filled boxes) showing a clear induction of active marks (H3K4me3 and H3K9Ac).

Another finding with potential implications in the late pathophysiology of the disease is the alterations of the promoter regions of IL-1β as well as the IL1 Receptor 2 (IL1R2). While the promoter of IL-1β is depleted of active marks (H3K4ac and H3K4me3, data not shown), both marks are accumulating at the IL1R2 promoter (Fig. 4). Remarkably, these results are in line with a very recent study in post-traumatic patients developing sepsis, which demonstrates a down-regulation of IL-1β as well as an up-regulation of IL1R2 gene expression and proposingg a suppression of innate immunity [25].

Sepsis might lead to changes in the expression of certain important transcriptional regulatory factors, resulting in changes in the modification pattern of downstream promoters. Interfering with these factors might be a promising therapeutic approach, for which already experimental evidence exists. By applying the synthetic histone I-BET, which mimics acetylated histones, pro-inflammatory gene expression is abolished during systemic inflammation [26]. In addition, the global pharmacological inhibition of HDAC activity by trichostatin A was found to protect mice from liver injury [27].

Our first results might be promising, but it is a long way to step out of the dark of applying treatment in a blackbox scenario towards the light of guided, surgical precise modulation of the epigenome. Before this might happen, we need to conduct further studies with larger sample sizes, and eventually including more histone modifications and especially more cell types—both hematopoetic and somatic. Moreover, as sepsis is a dynamic syndrome, we need to understand the spatiotemporal kinetic of epigenomic patterns and their functional consequences as well as differences depending on the underlying cause or focus of infection. Nevertheless, we believe that this bottom-up approach “from promoter to target” will raise new therapeutic options for the treatment of sepsis and other inflammatory diseases.

All together this study provides the first comprehensive ressource of the epigenomic changes occurring in CD14 ++ CD16- monocytes of patients suffering from sepsis. The genome-wide maps of three histone modifications indicate that a defined set of immunologically relevant genes undergo specific alterations of their associated histone modification patterns in human sepsis.

## Methods

Ethics Statement: All experiments were conducted according to the principles expressed in the Declaration of Helsinki, with approval of the ethics committee of the medical faculty of the Justus-Liebig-University of Giessen, Klinikstrasse 32, D-35385 Giessen, Germany (Approval number 155/12). Subjects provided written, informed consent on forms approved by the Institutional Review Board.

Cell separation: For monocyte separation 30 ml blood sample was taken and immediately processed from 2 patients suffering from sepsis and 4 healthy donors. Ficoll-based density gradient centrifugation was used to isolate peripheral blood mononuclear cells. To isolate CD14++ CD16- monocytes, magnetic cell sorting (autoMACS, Miltenyi Biotec) was conducted using CD16 MicroBeads (Miltenyi Biotec) to deplete CD16++ monocytes and CD14 MicroBeads (Miltenyi Biotec) to finally isolate CD14++ CD16- monocytes.

Purity of CD14++ CD16- monocytes was measured using flow cytometry after incubation with FITC anti-human CD14 Antibody (BioLegend).

ChIP protocol: ChIP was performed as described before [18]. CD14++ CD16- Monocytes were cross-linked for 10 min using formaldehyde (final concentration 1%) at 18°C. Fixation was stopped with glycine (final concentration 0.125 M). After lysis (10^6^ cells per 200 μl lysis buffer) sonication was performed using Branson sonifier (fragment size 150–600 base pairs) under constant cooling. Preparation of immunoprecipiation was conducted after seperation of 10% input using agarose A/G-beads (Calbiochem). Chromatin was mixed with antibodies for H3K4me3, H3K27me3 and H3K9ac at 4°C in a rotator for 3 hours. Different concentrations of salt wash buffer were to clear chromatin bead complex, before reverse Cross-linking (RNase A followed by 10% SDS/Proteinase K mix). Illustra GFX PCR DNA and Gel Band Purification kit (GE Healthcare Life Science) was used to purify DNA. DNA was eluted in pure H2O and quantitation of ChIP-DNA determined by Qubit Assay (Qubit Fluorometric Quantitiation; Life Technologies).

Library amplification: We used MicroPlex Library Preparation kit (Diagenode) for library amplification with the presence of SYBR Green in a real-time PCR machine as previously described [18]. AMPure XP beads were used for library purification. qPCR was used to validate the amplified product on selected target sequences.

Sequencing: Illumina HiSeq was used for sequencing of 4 barcoded libraries (input, H3K9ac, H3K4me3 and H3K27me3) for each patient and healthy donor on a single lane. Cluster generation was performed using the Illumina cluster station, sequencing on the Illumina HiSeq 2500 followed a standard protocol. The fluorescent images were processed to sequences using the Genome Analyzer Pipeline Analysis software 1.8 (Illumina). All sequencing data was deposited at NCBI’s gene expression omnibus (GEO) under the accession number GSE62908.

### ChIP-seq analyses and data visualization

Processing of ChIP-seq reads: ChIP-Seq reads were converted to fastq format and aligned to a precompiled hg19 reference index with BOWTIE [28]. Sequencing data was controlled for general quality features using FastQC. Unambiguously mapped and unique reads were kept for subsequent generation of binding profiles. All downstream analyses were done in R/BioConductor (http://www.bioconductor.org).

Differential promoter binding analysis: Read counts were determined for the promoters of all annotated hg19 RefSeq transcripts. The RefSeq transcriptional start sites (TSSs) were used to identify promoter intervals ranging from −1 to +1 kb around the TSS. For each promoter the number of reads mapping to the respective interval were extracted for patient and control samples. DESeq [29] was used to determine changes in occupancy when comparing patient and control samples and we ranked the genes accordingly. For the generation of the heatmap in Fig. 2 we summarized the log2(fold change)-values for all promoters that showed an absolute fold change greater than two-fold and a p-value smaller than 0.05 for at least one histone modification mark when comparing patient and control samples. Hierachical clustering was applied on a Euclidean distance matrix of the corresponding tabular data using R's *hclust* function using the "complete" method.

Visualization of binding profiles: After extension of reads, continuous coverage vectors were calculated and normalized per million reads to account for differential library sizes. Additonally the coverage vectors were smoothed in a 1000 bp window, quantile-normalized and subsequently converted into bigwig format for visualization in genome browsers. The coverage vectors were used to collect data in windows of different sizes spanning transcriptional start sites using the bigWigSummary tool [30] application. The binding data was binned across binding sites in 50bp windows and the mean was calculated at each position in order to generate cumulative binding profiles.

Identification of enriched GO terms: Clusters identified by hierarchical clustering as described above were used to identifiy over-reprasented GO-terms using DAVID [31].

### Statistics

Statistics were calculated by the Mann-Whitney signed Rank test or paired t-test using R.

## Supporting Information

S1 Fig Individual patients and controls show same systematic effects on histone modification levels at MHC gene promoters as identified in the grouped comparison. Boxplots shows z-scaled read counts of MHC genes for individual patients (#P1 and #P2: red boxes) and controls (#C1-#C4: blue boxes) for three modifications.
(TIF)

S1 Table Baseline characteristics of patients with sepsis. (XLSX)

S2 Table Complete list of genes with differential promoter coverage. Summary statistics describing differential association of histone modifications comparing patients and controls over 743 gene promoters with significant changes for at least one histone modification.
(XLSX)

S3 Table Gene list of “silent” and “active” cluster. (XLSX)
