# Supplementary figures and images for: Sepsis Induces Specific Changes in Histone Modification Patterns in Human Monocytes
Source: PLoS One. 2015 Mar 20;10(3):e0121748. doi: 10.1371/journal.pone.0121748 (PMC4368631; doi:10.1371/journal.pone.0121748)

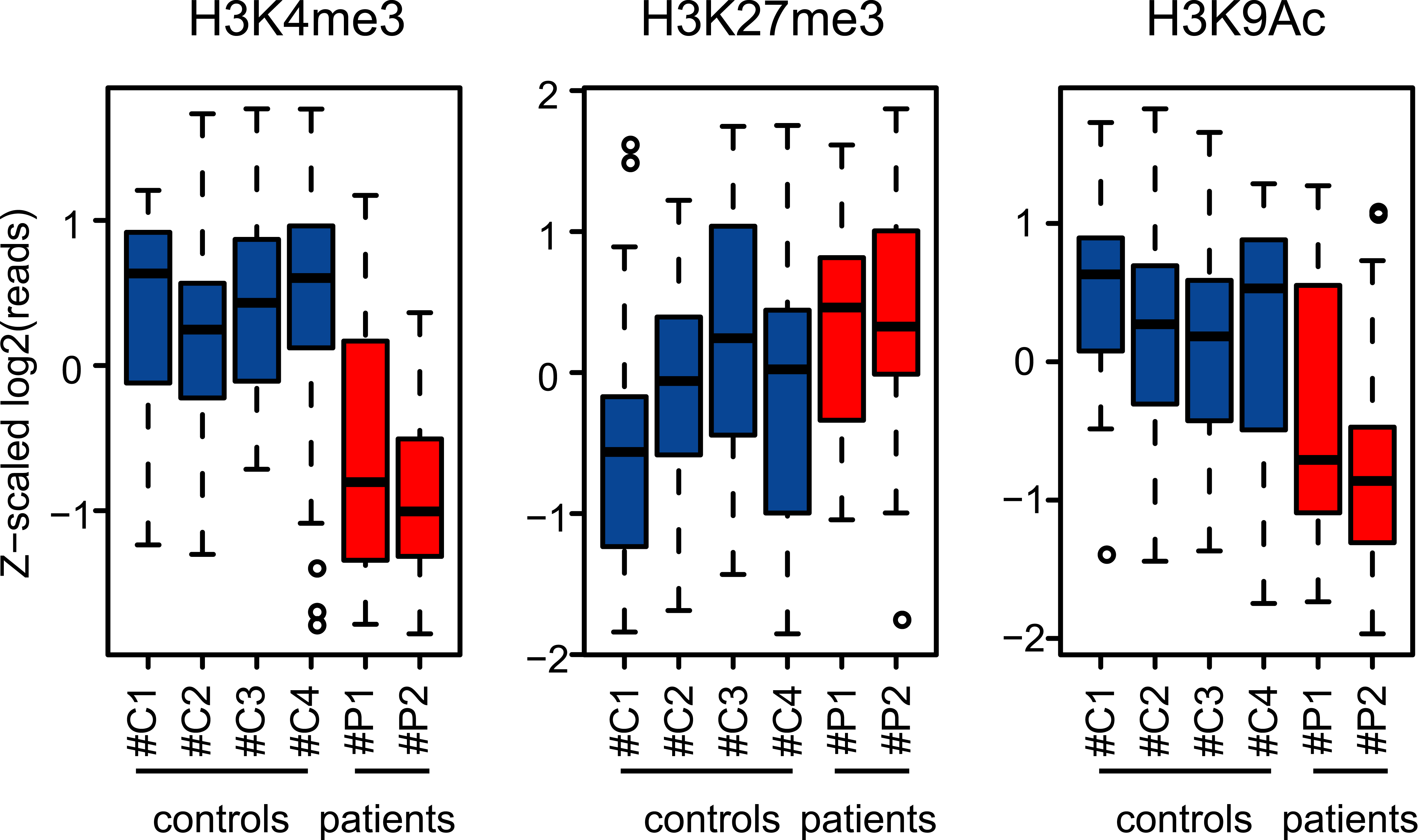

Supplement: S1 Fig — Boxplots shows z-scaled read counts of MHC genes for individual patients (#P1 and #P2: red boxes) and controls (#C1-#C4: blue boxes) for three modifications. (TIF) [file pone.0121748.s001.tif]
